# Supplementary material for: Extraction of Uranium in Nitric Media with Novel Asymmetric Tetra-Alkylcarbamide
Source: Molecules. 2022 Aug 27;27(17):5527. doi: 10.3390/molecules27175527 (PMC9457804; doi:10.3390/molecules27175527)
Supplement: Supplementary file 1 [file molecules-27-05527-s001.zip › molecules-1847422-supplementary V1-.pdf]

Extraction of uranium with novel tetra-alkylcarbamides

Qi Chen, Baole Li, Junli Wang, Haowei Zhu, Xiwen Chen, Yifu Hu, Jia Zhou, Xiang Li, Weifang Zheng, Taihong Yan*

China Institute of Atomic Energy, P. O. Box 275−26, Beijing, China, 102413.

*E-mail for the *corresponding author: yanth@ciae.ac.cn*

Supporting Information

CONTENTS

S1 Experiment

S1.1 Synthesis of the ligands

S1.2 ^{1}H-NMR spectrum of ligands

S1.3 ^{13}C-NMR spectrum of ligands

S1.4 High resolution Mass Spectrogram

S2 Results

S2.1 Extraction Kinetics Studies

S1 Experiment

S1.1 Synthesis of the ligands

Step 1: Dissolve 129 g of dibutylamine in 500 mL of carbon tetrachloride and stir for 30 min. Dissolve 118 g of triphosgene in 200 mL of carbon tetrachloride and slowly drop it into the dibutylamine solution. Heat the reaction to 90℃ for 6 hours. Spin dry the solvent and evaporate the intermediate at 120℃ under reduced pressure.

Step 2: (***L1***) Add 500 mL of tetrahydrofuran, 90 mL of triethylamine, and 183 mL of di-n-hexylamine to a 2000 mL three-necked flask, stir for 15 minutes at room temperature, add 172 g of Intermediate, and react for 12 hours at room temperature. Spin dry the solvent, add 500 mL ethyl acetate, wash 3 times with 300 mL saturated salt water, separate the organic phase, dry anhydrous magnesium sulfate, and spin dry to get ***L1***.

(***L2***) The synthesis procedure is basically similar to that of ***L1***, only replacing di-n-hexylamine with di-n-pentylamine.

S1.2 1H-NMR Spectrum of Ligands

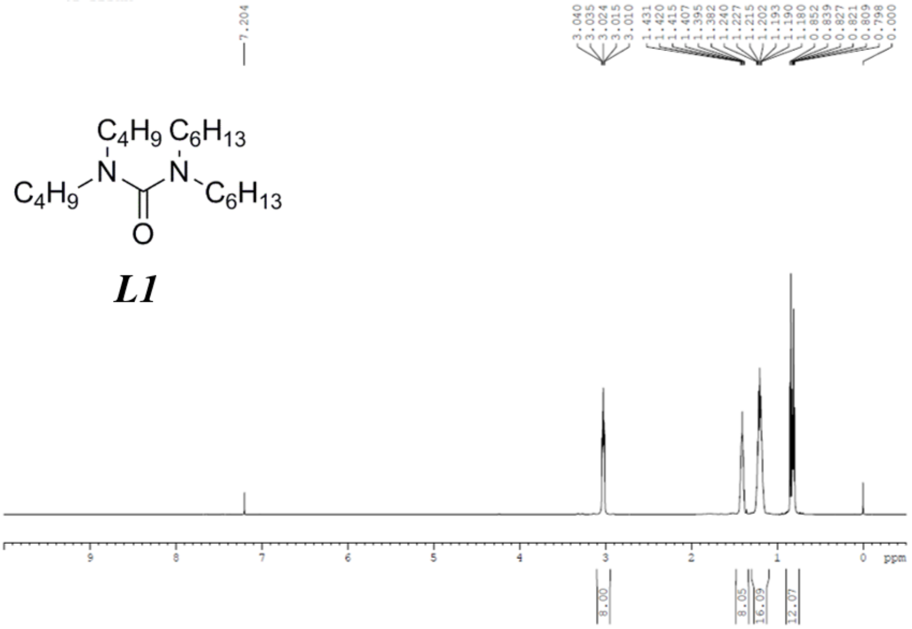

Figure S1. ^{1}H-NMR spectrum of ***L1***.

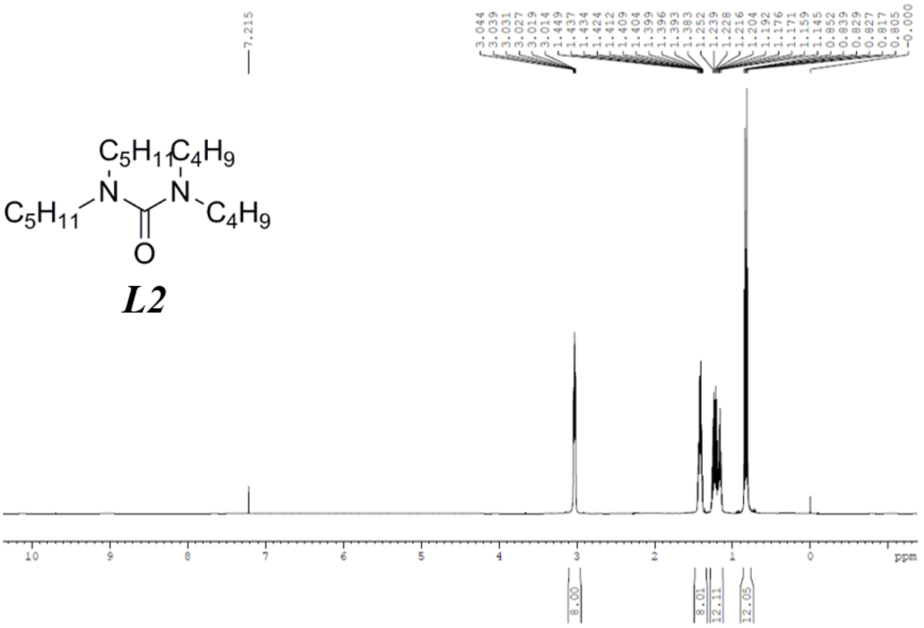

Figure S2. ^{1}H-NMR spectrum of ***L2***.

S1.3 ^{13}C-NMR Spectrum of Ligands

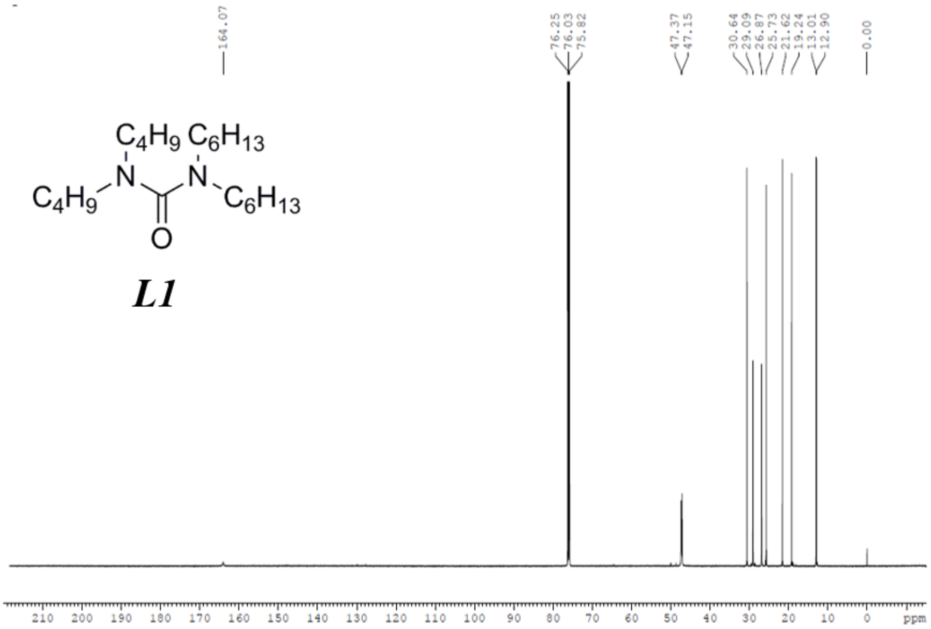

Figure S3. C-NMR spectrum of ***L1***.

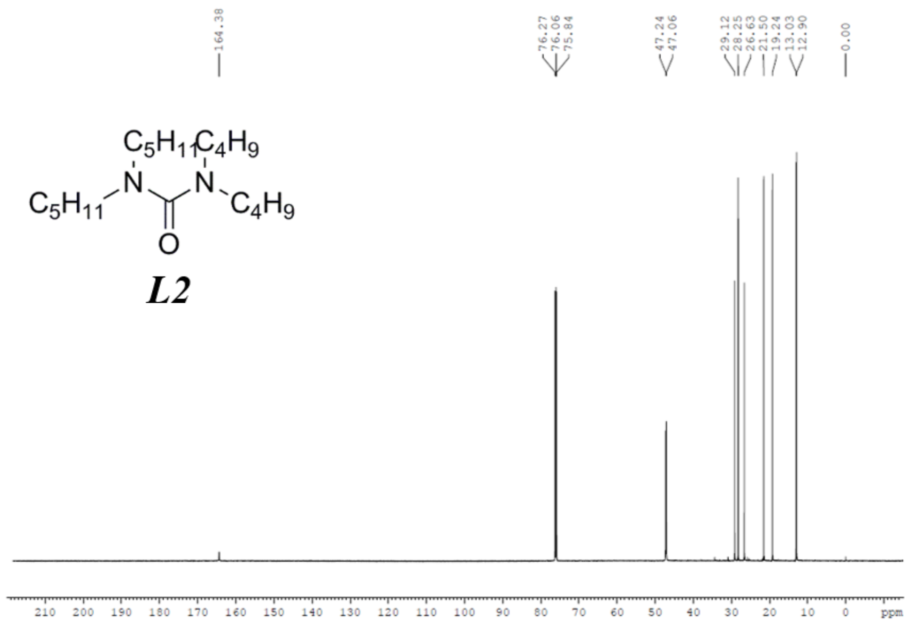

Figure S4. C-NMR spectrum of ***L2***.

S1.4 High resolution Mass Spectrogram

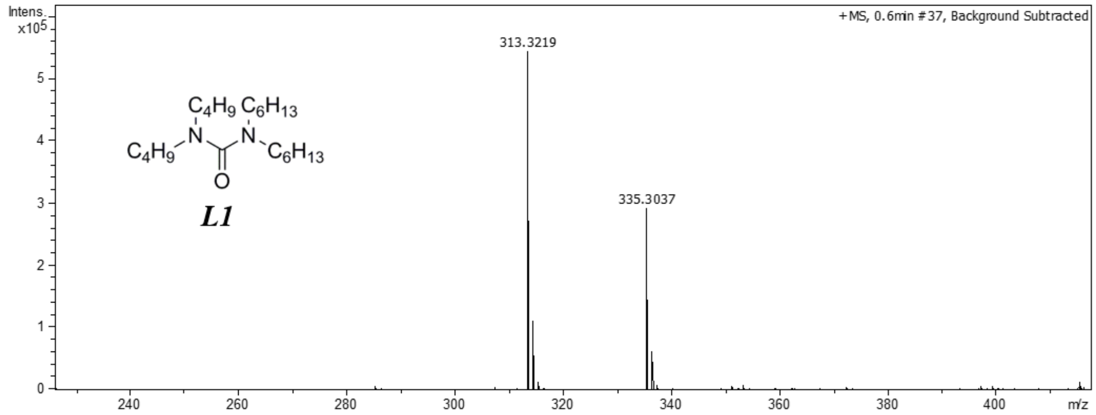

Figure S5. High resolution Mass Spectrogram of ***L1***.

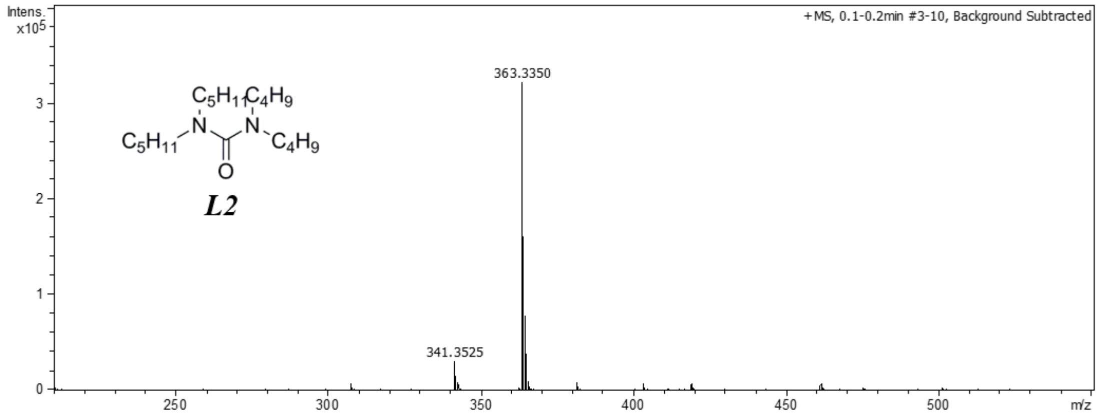

Figure S6. High resolution Mass Spectrogram of ***L2***.

IR spectra were recorded using a Shimadzu Iraffinity-1s FITR spectrometer. ^{1}H NMR spectra and ^{13}C NMR spectra were recorded using a Q.One Quantum-I Plus spectrometer. The chemical shifts (δ)

are reported in ppm. High resolution Mass Spectrogram were recorded by Agilent 1260 Infinity II-(Time of Flight)6230B.

1. **N,N-butyl-N',N'-hexylurea (*L1* : ABHU) :** ^{1}H NMR ($CDCl_3$, 600 MHz) δ 3.02 (m, 8H), 1.41 (m, 8H), 1.20 (m, 16H), 0.82(m, 12H) ppm. ^{13}C NMR ($CDCl_3$, 150 MHz) δ 165.4, 48.4, 48.0, 31.7, 30.1, 28.0, 26.8,22.6, 20.3, 14.0,13.9 ppm.IR (KBr): 2957, 2928, 2860, 1648, 1466, 1413, 1377, 1291, 1136, 1111, 727 cm^{-1}.

2. **N,N-butyl-N',N'-pentylurea (*L2* : ABPU) :** ^{1}H NMR ($CDCl_3$, 600 MHz) δ 3.12 (m, 8H), 1.50 (m, 8H), 1.29 (m, 12H), 0.92(m, 12H) ppm. ^{13}C NMR ($CDCl_3$, 150 MHz) δ 164.4, 47.2, 47.1, 29.1, 28.3, 26.6, 21.5, 19.2, 13.0, 12.9 ppm. IR (KBr): 2957, 2929, 2861, 1648, 1466, 1413, 1377, 1278, 1209, 1136, 941, 731 cm^{-1}.

S2 Results

S2.1 Extraction Kinetics Studies

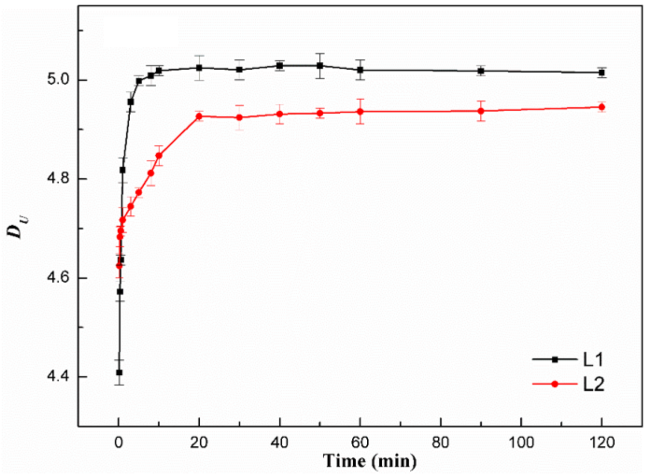

Figure S7 D_U by ***L*** in n-dodecane as a function of the contact time ($[HNO_3]_{ini}$ = 5 mol/L, $[UO_2^{2+}]_{ini}$ = 10 g/L, [***L***] = 0.5 mol/L).

The extraction kinetics of ***L*** were examined at first with the results displayed in Fig **S6**. The extraction contact time of the two ligands is found to be similar. Within 20 minutes, the extractions of U(VI) using the ***L***/n-dodecane system had reached equilibrium. The following liquid-liquid extraction tests were carried out for 30 minutes to assure full extraction of U(VI).
